# Designing Mixed Species Tree Plantations for the Tropics: Balancing Ecological Attributes of Species with Landholder Preferences in the Philippines

**DOI:** 10.1371/journal.pone.0095267

**Published:** 2014-04-21

**Authors:** Huong Nguyen, David Lamb, John Herbohn, Jennifer Firn

**Affiliations:** 1 School of Agriculture and Food Sciences, The University of Queensland, St Lucia, Australia; 2 Forest Industries Research Centre, The University of the Sunshine Coast, Sippy Downs, Australia; 3 Centre for Mined Land Research, The University of Queensland, St Lucia, Australia; 4 Faculty of Science and Technology, School of Earth, Environmental and Biological Sciences, Queensland University of Technology, Brisbane, Australia; Lakehead University, Canada

## Abstract

A mixed species reforestation program known as the Rainforestation Farming system was undertaken in the Philippines to develop forms of farm forestry more suitable for smallholders than the simple monocultural plantations commonly used then. In this study, we describe the subsequent changes in stand structure and floristic composition of these plantations in order to learn from the experience and develop improved prescriptions for reforestation systems likely to be attractive to smallholders. We investigated stands aged from 6 to 11 years old on three successive occasions over a 6 year period. We found the number of species originally present in the plots as trees >5 cm dbh decreased from an initial total of 76 species to 65 species at the end of study period. But, at the same time, some new species reached the size class threshold and were recruited into the canopy layer. There was a substantial decline in tree density from an estimated stocking of about 5000 trees per ha at the time of planting to 1380 trees per ha at the time of the first measurement; the density declined by a further 4.9% per year. Changes in composition and stand structure were indicated by a marked shift in the Importance Value Index of species. Over six years, shade-intolerant species became less important and the native shade-tolerant species (often Dipterocarps) increased in importance. Based on how the Rainforestation Farming plantations developed in these early years, we suggest that mixed-species plantations elsewhere in the humid tropics should be around 1000 trees per ha or less, that the proportion of fast growing (and hence early maturing) trees should be about 30–40% of this initial density and that any fruit tree component should only be planted on the plantation margin where more light and space are available for crowns to develop.

## Introduction

Over the past two decades, there has been rising interest in planting mixtures of tree species to establish plantations that provide multiple services including production and improved nutrient cycling and also to provide more biodiversity at the landscape level [1]. Mixed-species plantations have the potential to generate a variety of forest products, as well as a range of ecosystem services [2]–[4]. Mixed species plantations are often established using just two or three species but sometimes far more diverse mixtures have been used which include representatives of a variety of successional stages [5].

A major issue facing those wishing to establish mixed species plantations is that knowledge of the silvicultural attributes of most species is usually so limited that it is difficult to predict how these will grow when planted into novel combinations [6]. The growth strategies of species growing in natural forests can provide an indication of the role they might play in a plantation including whether they are shade-intolerant, a canopy dominant species or whether they can grow in sub-canopy strata. The interactions among species will strongly influence the productivity of mixtures [7], [8]. Species attributes that could be used as possible indicators of the performance of species grown in a mixed-species stand include shade tolerance/intolerance, height growth rate, crown structure, foliar phenology, and root depth and phenology [9], [10]. Combinations of species with complementary traits can reduce competition and allow for the most efficient use of limiting resources like water, nutrients and light in plant communities [6].

Identifying complementary species is a difficult task, particularly in the tropics when native tree species are preferred, as there is a limited knowledge of growth strategies of most native species. A number of challenges are likely to take place in newly established mixed-species stands because of competition and differences in the species growth rates. Trees grown in mixed species stands can sometimes suffer both intra-specific competition and inter-specific competition. Both of these are influenced by tree density [11]–[13]. High initial planting density may facilitate early site capture, reduce weed control, improve form and lower stem taper [14]. On the other hand, higher densities mean costs are greater because more seedlings are needed and stand thinning is necessary at an earlier stage of stand development. This problem may not be as great as it seems if the thinned trees have some economic value and there are usually some fast growing species that can be harvested at an early age.

The Rainforestation Farming plantations in the Philippines present an ideal opportunity to explore basic questions concerning forest dynamics, optimal planting densities and human-use patterns of tropical polycultures [15]–[19]. We hypothesise that these mixed species plantations could balance the ecological attributes of species with landholder preferences in the Philippines. Here, over three time periods, we measure changes in the structure and species composition of these mixtures. We consider the following questions:

- How has the composition and structure of these mixed-species plantings changed over time?
- How have patterns of species loss (due to mortality and harvesting) and recruitment been affected by the design of these species mixtures?
- What are the implications for the future design and management of tropical polycultures used by smallholders?

## Materials and Methods

### Ethics Statement

The study was permitted by the owners of the lands (for more details see Table 1) to be conducted in 18 sites. It was not possible to sample from all 28 sites that were established under the Rainforestation Farming system because several plantations had been detrimentally affected by fire, harvesting, clearing for other agricultural activities; because access was not granted by the land owners; or did not meet minimum requirements for measurements (e.g. trees greater than 5 cm diameter).

**Table 1:** Site characteristics and planting history of 18 of the mixed-species sites in Leyte Province, the Philippines*.

Site	Site location	Year planted	Area (ha)	Soil type	Topography	No. of plots
02	Marcos, Baybay	1995	0.61	Clay loam	Slightly to moderately rolling	2
03	Catmon, Ormoc	1998	1.4	Clay loam	Flat	12
04	Patag, Baybay	1998	1.0	Clay loam	Slightly rolling	5
05	Cienda, Baybay	1996	0.9707	Clay to clay loam	Flat	9
06	Pomponan, Baybay	1997	0.38	Clay loam	Slightly rolling	2
07	Punta, Baybay	1996	5.442	Limestone	Moderately rolling	9
08	Maitum, Baybay	1996	0.478	Clay loam	Slightly to moderately rolling	2
09	Mailhi, Baybay	1996	3.22	Clay to clay loam	Slightly to moderately rolling	10
10	Vila Solidasidad, Baybay	1995	0.4377	Clay	Flat	4
11	Maitum, Baybay	1996	0.4686	Limestone	Moderately rolling	2
12	Maitum, Baybay	1996	0.9862	Limestone	Slightly rolling	2
13	Maitum, Baybay	1996	0.2518	Clay loam	Moderately rolling	2
14	Pomponan, Baybay	1996	0.9518	Clay loam	Slightly to moderately rolling	2
15	Pomponan, Baybay	1997	0.438	Clay	Moderately rolling	2
16	Pomponan, Baybay	1997	0.41	Clay loam	Moderately rolling	2
17	Pomponan, Baybay	1999	0.8475	Sandy loam	Flat	2
19	Licoma, Ormoc	2000	0.25	Clay loam	Moderately rolling	2
22	Milagro, Ormoc	1996	1.5	Clay loam	Flat	7

*Milan et al. 2004.

### Study Area

The study was conducted in Leyte province, which is one of two provinces located in Leyte Island (Figure S1 in File S1). This is the eighth largest island in the middle of the Philippines archipelago and covers about 800,000 ha [20], [21]. Leyte Province has a humid monsoon climate and the average rainfall in the study area for the years 1980−2000 was 2,686 mm with an annual variation of between 1,775 mm in 1987 and 3,697 mm in 1999 [22]. Although there is no pronounced dry season, the region experiences its lowest rainfall of less than 100 mm between March and May [23]. Dry periods of several months duration with rainfall of less than 100 mm can sometimes occur as was the case during the ‘El Nino’ year of 1993, the year in which the project commenced. The average annual temperature is 27.5°C and ranges from 26.3 to 28.7°C. The relative humidity is always high and the average monthly level for the years 1980−2000 ranged from 75.1% in March to 80.1% in October [22]. The soils are derived from volcanic parent material and were strong acidic with a pH 4.1–4.9 [24]. The natural vegetation in the region is a species-rich, lowland dipterocarp forest but natural forest now only remains on the less accessible slopes of the Leyte cordillera [25].

A unique polyculture reforestation program was started in the Philippines in 1992 called the Rainforestation Farming system [26]–[29]. It involved 28 small-scale mixed-species plantations on private farms on Leyte Island. These plantings were established to provide smallholders with a form of reforestation that generated income from an early age while also providing the benefits of increased biodiversity in the highly cleared and mainly agricultural landscape. The intent was to create resilient and sustainable forms of reforestation that would also be financially attractive to local farmers [27], [28]. The program focussed on native species and used a large number of species to establish mixtures to resemble a few natural forests in the region. In response to demands by landholders some exotic species were also used in the mixtures. The system used approximately 100 endemic pioneer and shade-intolerant tree species, longer-lived species of Dipterocarp, fruit trees and a limited number of exotic timber species. These were planted to create a series of small scale plantations in the average area of about 1 ha [29].

At the beginning of the Rainforestation Farming project, species assessed as shade-intolerant pioneers were planted in a spacing of 2 m×2 m to provide an environment for supposedly shade-tolerant timber and fruit tree species to be established in the following year. These were inter-planted at a general spacing of 2 m×1 m [28]. The estimated density at planting time was about 5000 trees/ha at sites. From 7 to 40 species were planted at each farm. The aim was to create a three storied structure with pioneer and shade-intolerant trees in the upper canopy layer, shade-tolerant trees and fruit trees in the second storey and shade-tolerant crops in the lowest layer. Supposedly there would be roughly equal numbers of shade-tolerant and shade-intolerant species. In fact the numbers and identity of species planted at each farm were not consistent but depended upon seedling availability at the time, and the preferences of the local landholder. As a consequence detailed records of plantings at each farm were not kept. Sites were developed over a period of several years from 1995 to 2000. Further details of the Rainforestation Farming methodology are given by Kolb [22].

### Data Collection and Analysis

Data were collected from 80 plots distributed across 18 of the mixed-species plantations (Table 1). These plots have also been used in a recent biodiversity-productivity study [18]. The first measurement of species composition and stand structural characteristics was undertaken from February to March 2006 when the trees were aged between six and 11 years. Measurements of trees and site properties were collected from randomly located circular plots with a radius of 5 m (78 m^2^ area) within the plantations. The number of plots sampled at each farm ranged from 1 to 12 plots on the size of the farm’s plantings, with the number being determined by the size of site so that the sampling area occupied at least 5% of plantation [30], [31]. All plots and trees within them were permanently marked in the field. In each plot, all trees were counted and identified to the species level and the height (H) and diameter at breast height (dbh) were measured. Each plot contained at least seven trees greater than 5 cm in diameter. All the plots were re-measured during 2008 and again in 2012. On each occasion new recruits were identified and measured and tree deaths were recorded. A distinction was made between deaths and by harvesting (i.e. evidence in the form of stumps) and natural tree deaths.

The species were grouped into two categories based on provenance (i.e. native species and exotic species) and three categories based on ecological types. These included shade-intolerant species (pioneer as well as longer lived secondary forest species), shade-tolerant species (species from the Dipterocarpaceae plus other shade tolerant species) and fruit tree species. All of the shade-tolerant species but especially the Dipterocarps are regarded locally as having highly valued timbers.

Annual rates of mortality and recruitment were calculated as the mean annual proportion of lost trees or new recruits using the total number of stems at the time of the first measurement as the base line.

The estimated times needed for species growing in Rainforestation stands to reach threshhold sizes of 10 cm and 30 cm were calculated based on relative growth rates of the 32 common species across sites.

The Importance Value Index (IVI; see Table 2) was used to assess the importance of different species in each plantation [32]–[36].

**Table 2:** Equations to calculate Important value index (IVI) of species.

Index	Equation
Importance value index (IVI)	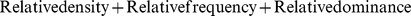
Relative density	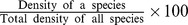
Relative frequency	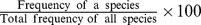
Relative dominance	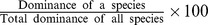
Density	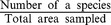
Frequency	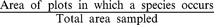
Dominance	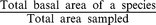

One-way ANOVA test was used to compare stand densities and the Importance Value Indices between the three measurements. The differences in tree loss and recruitment between species groups were also analysed using a one-way ANOVA combined with LSD post-hoc tests for all pairwise comparisions between group means.

Statistical analysis was undertaken using SPSS 21 and Excel 2010 and data was visually presented using SigmaPlot 12.3.

## Results

### Species Compositional Changes

A total of 76 canopy species of trees were recorded at 80 plots distributed across the 18 sites at the time of the first measurement in 2006 (Table 3). These represented 33 families and 58 genera. They included 43 shade-intolerant species, 8 shade-tolerant non-Dipterocarp species, 11 Dipterocarp species (all shade-tolerant) and 14 fruit tree species. Of these, 57 are native species and 19 are exotic species.

**Table 3:** Changes of species composition of canopy trees in the Rainforetation plantations over time.

Category	Remaining number of species		
	2006	2008	2012
Shade-intolerant species	43	38	35
Shade-tolerant species	19	18	19
Fruit-tree species	14	12	11
Native species	57	50	49
Exotic species	19	18	16
Total	76	68	65*

*includes a new species recruited from outside plot.

Each of the different species types were present at most sites, but the proportion of trees represented by the different types of species varied: exotic species represented 36% of all trees, while shade intolerant species represented 78% of all trees. Fruit trees were present in only 21 of the 80 plots and represented 8% of all trees.

Overtime, we found the number of species present decreased from 76 in 2006 to 65 in 2012 (Table 3). The species lost included eight that were shade-intolerant, three fruit tree species and one species of Dipterocarpaceae. On the other hand an additional species (*Strombosia philippinensis*) was recruited from trees growing outside the plots in the period between 2008 and 2012 and grew up to exceed the 5 cm dbh size threshold above which trees were assessed.

### Changes in Stand Density Caused by Mortality and Harvesting

**Figure 1:**
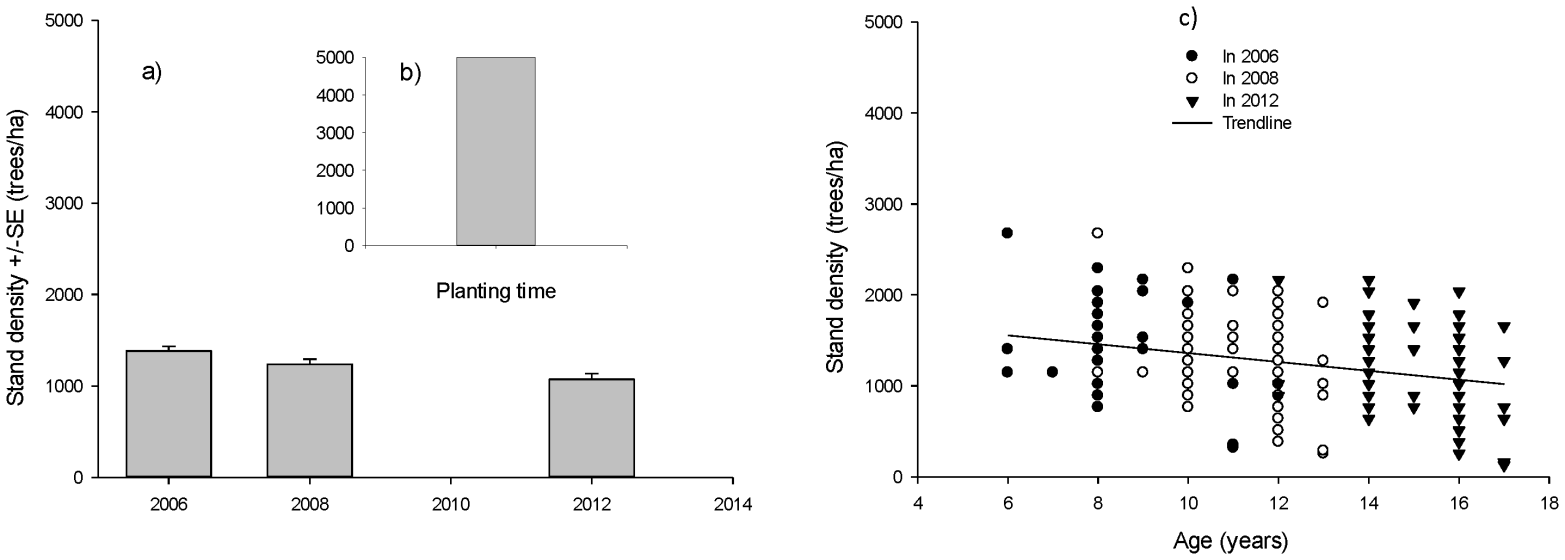
Changes in stand density of Rainforestation plantations over time. Mean stand density at measurements (a), estimated stand density at the planting time (b), and stand density of plots at different ages (c).

The average stand density across all sites decreased from an estimated planting density of 5000 trees ha^-1^ to 1383±50 trees ha^−1^ at the time of first measurement in 2006 when the sites were aged between six and 11 years. From 2006 the average stand density then decreased further to 1145±56 trees ha^−1^ in 2012 (Figure 1). These changes were not uniform and density was constant in 19 plots (24% total plots) during the whole period over which measurements were made. The average site stand density differed significantly over time (F_18_ = 4.720, p = 0.013).

**Figure 2:**
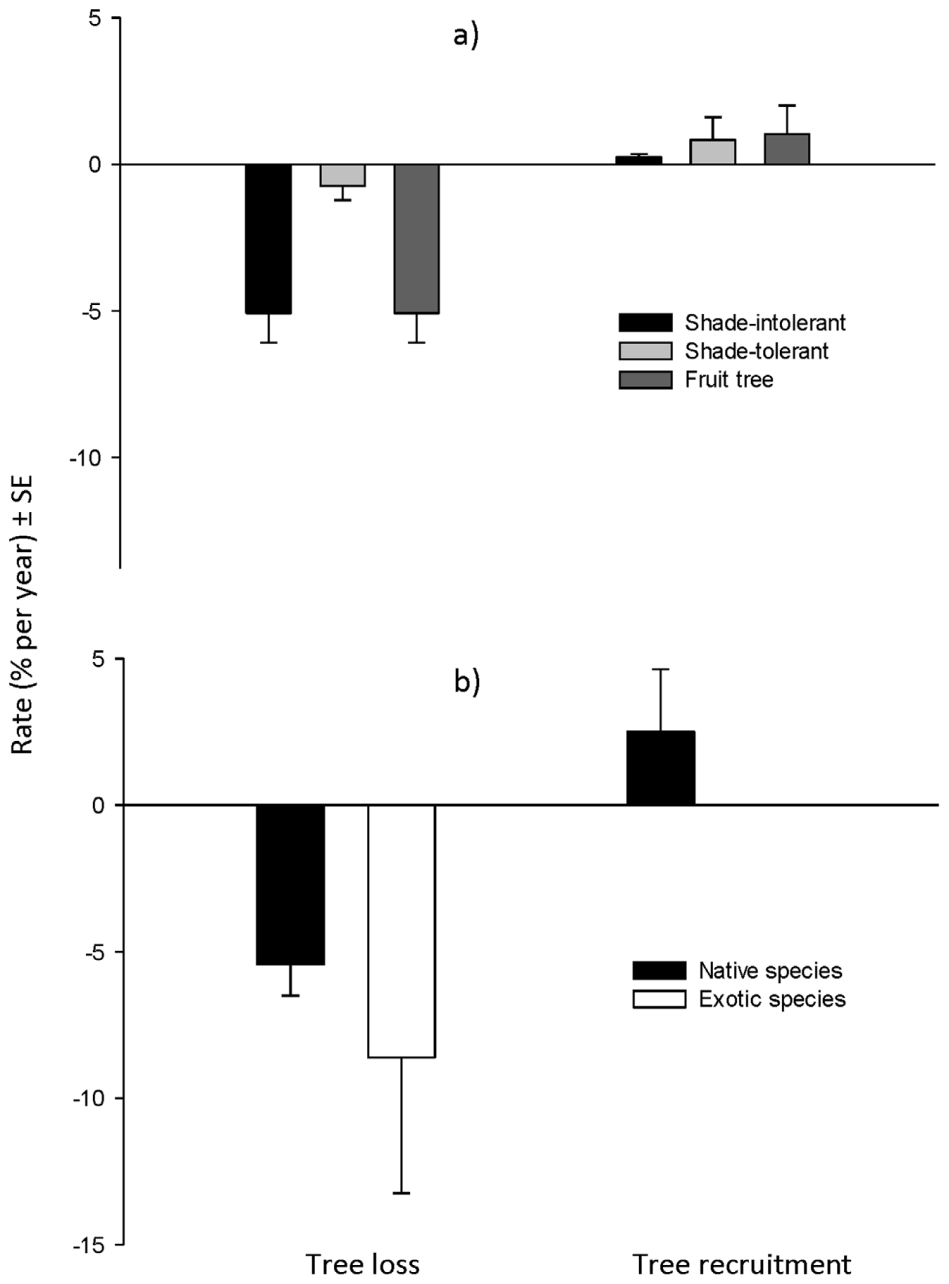
Annual rates of mortality and recruitment at sites during the period of 2006–2012. In categories of species ecology (a) and in categories of species provenance (b).

**Figure 3:**
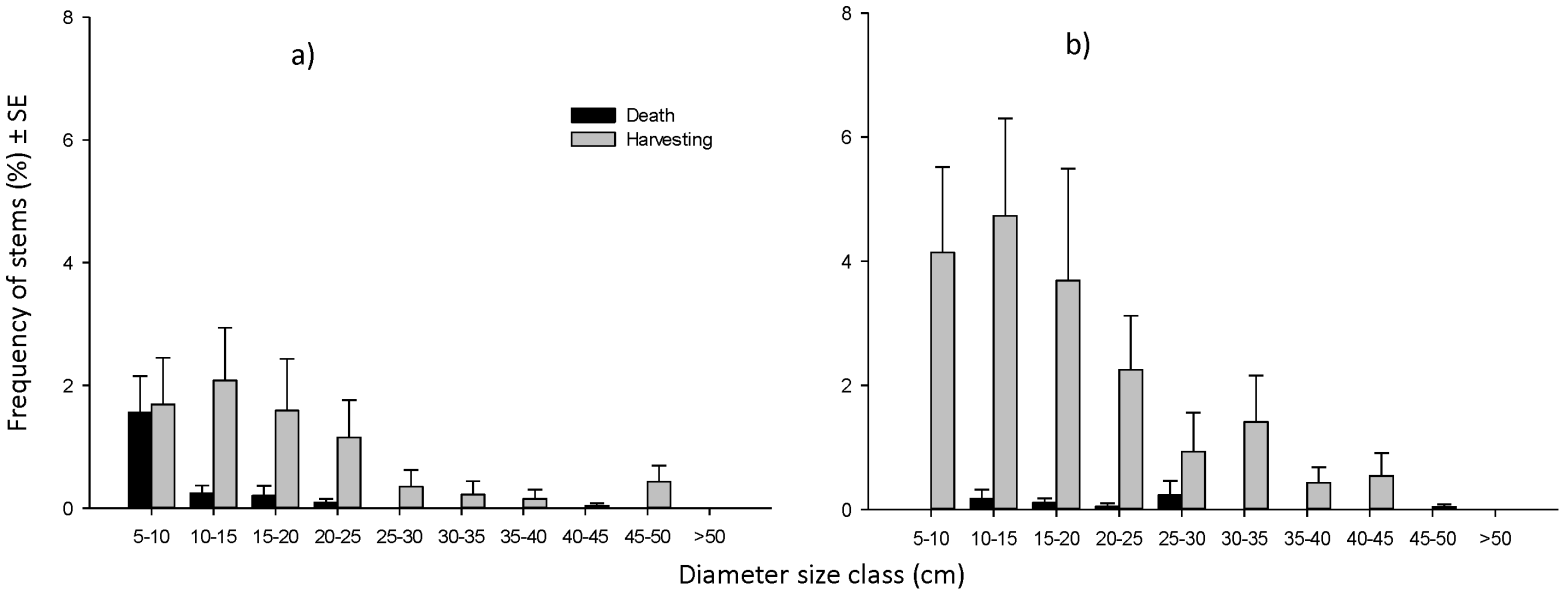
Size class distributions of lost trees (death and harvesting) at the Rainforestation sites. In the periods of 2006–2008 (a) and 2008–2012 (b).

Part of the decline was due to between-tree competition but some was also caused by tree harvesting by farmers. Loss due to competition (i.e. mortality) over the period differed between the various categories of species. Overall, shade-intolerant species and fruit trees lost around 5% of trees each year but only 0.7% of the shade-tolerant trees were lost (Table 4). Around 5.4% and 8.6% of individuals of native and exotic species respectively were lost each year due to competition and harvesting (Table 4, Figure 2). There was no significant difference in the loss of stems between native and exotic species (F_18_ = 0.450, p = 0.507) but a difference between functional groups of shade-intolerant, shade-tolerant and fruit trees (F_18_ = 3.111, p = 0.054) across sites. We found that the shade-tolerant group had lost significantly fewer stems than the other groups (p = 0.030 and 0.043 between shade-tolerant and shade-intolerant, and between shade-tolerant and fruit trees, respectively). We found no significant difference in terms of losing individuals between shade-intolerant species and fruit trees (p = 0.881). Most of the deaths occurred in trees less than 10 cm dbh although a few larger trees had also died by the time of the final measurement (Figure 3). Most of the losses occurred in smaller size classes and were more common in the 2006–2008 period than in the 2008–2012 period (Figure 3).

**Table 4:** Mortality and recruitment of species groups in Rainforestation plantations over time during the period of 2006–2012.

Category	Proportion of trees	
	Mortality (% per year)± SE	Recruitment (% per year)± SE
Shade-intolerant species	5.08±1.01	0.24±0.11
Shade-tolerant species	0.73±0.49	0.83±0.78
Fruit-tree species	4.79±2.11	1.03±0.98
Native species	5.43±1.08	2.51±2.13
Exotic species	8.62±4.63	0
New species in plots (but plantedspecies in farms)		0.004±0.004
Total	4.89±1.01	0.49±0.32

Evidence of harvesting and the identity of these trees could be seen in the form of old stumps. Harvesting commenced when the trees were in the 5–10 cm dbh class but harvesting was common in trees up to around 25 cm dbh (Figure 3). During the six years, 20.4% of all trees >5 cm dbh recorded in 2006 were harvested. The smaller sized trees harvested belonged to 38 species of all shade-intolerant, shade-intolerant species and fruit trees while the larger trees belonged to only 11 shade-intolerant species (e.g. *Melia dubia, Gmelina arborea*, *Leucaena leucocephala, Terminala macrocarpa* and *Swietenia macrophylla*) and only one individual of fruit tree (i.e. *Sandroricum koetjape*). Some trees of these species were also harvested at dbh < = 25 cm. The other common species that were harvested at dbh <25 cm included *Vitex parviflora*, *Gymnostoma rumphianum*, *Artocarpus heterophyllus*, *Samanea saman*, *Pterocymbium tinctorium* and *Tectona grandis*.

Most of trees which were died due to competition or disease had dbh smaller than 25 cm, including shade-intolerant, shade-tolerant and fruit trees; only one death of decayed tree of *Melia dubia* had dbh around 30 cm.

In contrast to these losses, a number of trees were also added to the stands as trees grew larger and exceeded the 5 cm dbh size class. These were found in 23 of the 80 plots. At the time of the first measurement in 2008 trees belonging to 9 species had been added to the stands and by 2012 trees from 20 native species were being added. They included Dipterocarp species (e.g. *Parashorea plicata*, *Shorea contorta*, *Hopea malibato*, *Hopea plagata*) and some individuals of other shade-tolerant, shade-intolerant and fruit trees. There were no exotic species found in the recruitment (Table 4, Figure 2).

### Growth Rates

By 2006 about 2% of all trees exceeded 30 cm dbh but this has increased to 6.7% by 2012 (see Figure S2 in File S1). The results showed that three shade-intolerant species (i.e. *Leucaena leucocephala*, *Melia dubia* and *Gmelina arborea*) could reach the dbh threshold of 30 cm by age 10 years. Most these species could achieve 30 cm dbh before 20 years old of planting. The estimated time it takes for particular species to reach a harvestable age is shown in Table 5.

**Table 5:** The estimated time needed for species growing in Rainforestation Farming stands to reach threshold sizes for firewood (10 cm dbh) and lumber (30 cm dbh).

Species	Provenance	Estimated time (years) to reach	
		10 cm dbh (Mean ± SD)	30 cm dbh (Mean ± SD)
**Shade-intolerant species:**			
Ipil-Ipil (*Leucaena laucocephala)*	Exotic	3.3±1.0	9.8±3.1
Bagalunga (*Melia dubia*)	Native	5.1±2.4	15.2±7.3
Gmelina (*Gmelina arborea*)	Exotic	5.9±3.5	17.6±10.5
Taluto (*Pterocymbium tinctorium*)	Native	7.0±2.6	20.9±7.7
Teak (*Tectona grandis*)	Exotic	7.5±3.6	22.6±10.8
Santol (*Sandoricum koetjape*)	Native	7.7±3.0	23.2±8.9
Mt Agoho (*Gymnostoma rumphianum*)	Native	7.8±2.8	23.5±8.3
Kalumpit (*Terminalia macrocarpa*)	Native	7.9±3.9	23.7±11.6
Raintree (*Samanea saman*)	Exotic	7.9±5.0	23.7±15.1
Dao (*Dracontamelon dao*)	Exotic	8.6±3.7	25.7±11.0
Nangka (*Artocarpus heterophyllus*)	Native	8.9±2.7	26.7±8.2
Thailand acacia (*Senna siamea*)	Exotic	9.3±2.5	28.0±7.4
Mahogany (*Swietenia macrophylla*)	Exotic	9.3±4.2	27.9±12.6
Molave (*Vitex parviflora*)	Native	9.4±3.1	25.2±9.3
Antipolo (*Artocarpus blancoi*)	Native	9.9±4.4	29.8±13.3
Bitanghol sibat (*Calophyllum lancifolium*)	Native	10.4±3.4	31.2±10.3
Narra (*Pterocarpus indicus*)	Native	10.6±3.7	31.7±11.2
Hindang laparan (*Myrica javanica*)	Native	10.9±4.7	32.6±14.2
Lanipga (*Toona ciliate*)	Exotic	10.3±2.8	30.9±8.5
Malakawayan (*Podocarpus rumphii*)	Native	18.2±4.2	54.7±12.5
**Shade-tolerant species:**			
Mayapis (*Shorea palosapis*)	Native	5.3±1.6	16.0±4.9
Tangeli (*Shorea polysperma*)	Native	6.5±2.7	19.4±8.1
Apitong hagakhak (*Dipterocarpus kunstleri*)	Native	8.0±3.6	24.1±10.8
Marang banguhan (*Artocarpus odoratissimus*)	Native	8.3±3.0	24.8±9.0
Bagtikan (*Parashorea plicata*)	Native	8.7±3.8	26.2±11.4
Rambutan (*Nephelium lappaceum*)	Native	8.8±2.4	26.3±7.2
Cacao (*Theobroma cacao*)	Exotic	8.9±2.6	26.7±7.9
White lauan (*Shorea contorta*)	Native	8.9±3.7	26.8±11.1
Almaciga (*Agathis philippinensis*)	Native	9.5±2.9	28.6±9.5
Durian (*Durio zibethinus*)	Exotic	9.8±3.7	29.5±11.2
Yakal saplungan (*Hopea plagata*)	Native	10.4±3.2	31.2±9.6
Yakal kaliot (*Hopea malibato*)	Native	12.0±2.7	35.9±8.2

### Importance Value Index

**Figure 4:**
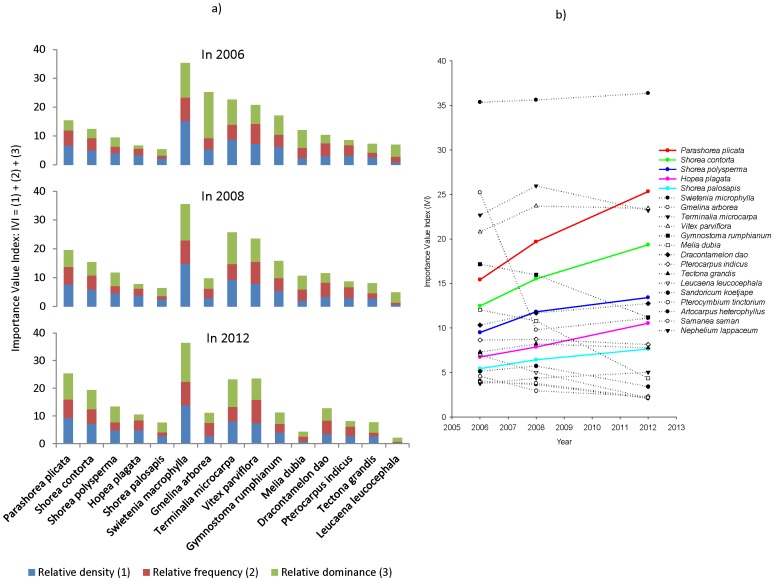
Importance Value Indices (IVIs) of the 15 common species with the highest IVI in rainforestation plantations in period of 2006–2012. IVI at measurements (a) and changing trend of IVI between 2006 and 2012 (b).

Although Importance Value Index of species was not significantly different between measurements across sites for all species (F_77_ = −1.1E-13, p>1), mortality and early harvesting by landholders caused a change in the Importance Value Index of some species in specific plantations (Figure 4). At the time of the first measurement shade-intolerant species had the highest Importance Value Indices due to their density, relative common and uniform distribution and their faster-growth (e.g. *Swietenia macrophylla*, *Gmelina arborea*, *Terminalia macrocarpa*, *Vitex parviflora* and *Gymnostoma rumphianum*). However, their Importance Values declined because of harvesting and the shade-tolerant species gradually acquired greater Importance Values (e.g. *Gmelina arborea*, *Gymnostoma rumphianum*, *Melia dubia* and *Leucaena leucocephala*) (Figure 4 and Figure S3 in File S1).

## Discussion

Our results indicate that the Rainforestation Farming plantations are a highly dynamic system. Changes in stand structure and species composition have been brought about by a combination of anthropogenic factors and natural mortality. It appears that the harvesting of trees by farmers was carried out in an entirely opportunistic way according to their particular circumstances; it was not based on silvicultural prescriptions that specified the timing or intensity of tree removals. This means it is unlikely that production was optimised in the way that managers of industrial plantations usually seek to achieve. On the other hand, this was exactly the intention of the designers of the Rainforestation Farming system; optimal productivity has been traded-off for the sake of flexibility. Mortality has been hastened by the very high initial planting densities. Not surprisingly this has mostly occurred amongst the shade-intolerant species. The net effect of these two processes has meant that over the six years of the study, slower growing native species have become more dominant while faster growing (mainly shade-intolerant) species have become less dominant. At this point it is not clear how these stands should be now managed because the timing of any future harvesting (i.e. financial benefit to landholders) is difficult to specify. It is likely that farmers will continue to remove trees once they reach some threshold size chosen by the landholder to suit their purpose irrespective of the value of the timbers or market for the logs.

Fruit trees were added to the species mixtures in the expectation that once mature, they would yield a large annual crop of fruit. Experience has shown however that this has not occurred even though some fruit trees are now more than 15 years old. Typically fruit trees require wide spacing and full sun to bear productively [15], [35]–[40]. But in the Rainforestation Farming design they were incorporated into a closely spaced system and were quickly shaded by faster growing pioneer species. As a consequence there appears to be high mortality of fruit trees. There are reports of some fruit harvesting at some sites by Milan et al. [26] but overall productivity has been very low. A better outcome might have been achieved by plantings fruit trees along plantation edges where they would receive more light and be easier to harvest.

Based on this experience we suggest a modified set of silvicultural prescriptions for smallholder and community tree plantations in this part of the Philippines, along with elsewhere in SE Asia. The suggestions are outlined in Table 6 and show recommended planting densities and constituent species. The planting density is set at around 1100 trees per ha rather than the 5000 trees per ha used in the Rainforestation Farming plantations. This reduces the cost of buying and planting seedlings and, while there may be slight increase in the time needed for weed control until the seedlings are established, this cost should be modest. The numbers of species used is reduced from that originally established in Rainforestation Farming plantings (at least 76 species assessed in the plots in this study) to between 11–23 species. This is because the type of species (i.e. fast growing and/or exotic versus slow growing/native) drives productivity in these plantings rather than increased biodiversity [18].

**Table 6:** Recommended design for smallholder tree plantations in Leyte, Philippines based on performance of Rainforestation Farming plantations.

Product	Time ofthinning (yrs)	Number ofspecies	Density(trees/ha)	Typical species
Firewood	6–10	3–5	450	Gmelina (*Gmelina arborea*)
				Bagalunga (*Melia dubia*)
				Ipil-Ipil (*Leucaena laucocephala*)
				Raintree (*Samanea saman*)
				Mt agoho (*Gymnostoma rumphianum*)
				Agoho (*Casuarina equisetifolia*)
				Thailand acacia (*Senna siamea*)
Pole	8–12	2–3	200	Gmelina (*Gmelina arborea*)
				Mahogany (*Swietenia macrophylla*)
				Kalumpit (*Terminalia macrocarpa*)
				Dao (*Dracontamelon dao*)
				Narra (*Pterocarpusindicus*)
				Mt agoho (*Gymnostoma rumphianum*)
Fast-growing timber	14–18	3–5	250	Gmelina (*Gmelina arborea*)
				Mahogany (*Swietenia macrophylla*)
				Teak (*Tectona grandis*)
				Mayapis (*Shorea palosapis*)
				White lauan (*Shorea contorta*)
				Almaciga (*Agathis philippinensis*)
				Tangeli (*Shorea polysperma*)
				Bagtikan (*Parashorea plicata*)
Slower-growing timber	>20	3–10	200	Yakal saplungan (*Hopea plagata*)
				Yakal kaliot (*Hopea malibato*)
				Malakawayan (*Podocarpus rumphii*)
				Molave (*Vitex parviflora*)
				Narra (*Pterocarpus indicus*)
				Malapanau (*Dipterocarpus kerrii*)
				Tangeli (*Shorea polysperma*)
				Apitong (*Dipterocarp grandiflorus*)
				Apitong hagakhak (*Dipterocarpus kunstleri*)
				Dalingdingan (*Hopea dalingdingan*)
				Red lauan (*Shorea negrosensis*)
				Bitanghol (*Calophyllum blancoi*)
				Bitanghol sibat (*Calophyllum lancifolium*)
Total		11–23	1100	Fruit trees: Durian (*Durio zibethinus*), Mango (*Mangifera indica*), Rambutan (*Nephelium lappaceum*), Nangka (*Artocarpus heterophyllus*)

The identity of these species has been based on their performance in the field (See Herbohn et al. [15] for the initial assessment) and the prospective markets likely to be available to farmers in this region. They include fast-growing species currently preferred by most farmers as well as slow-growing species likely to generate higher value timbers in the long term [26]. As was the case in the original Rainforestation Farming design, the shade intolerant species will be planted one year before the other species to facilitate their establishment. The intent of these prescriptions is to provide a steady flow of trees of sufficient size and with properties making them suitable for various markets. A significant proportion of the species planted are destined quickly capture the site and then be removed at an early age (6–10 years) in order to generate an income. This harvest will also act as a thinning to prevent the stands stagnating and to hasten the development of larger and more valuable residual trees. Most of these will be faster-growing species such as *Gmelina arborea, Casuarina equestifolia* or *Acacia* sp but some slower-growing species having trees with poor form might also be felled at this time as well. *Casuarina equisetifolia* was not found in our plots but observed in surrounding areas, we, therefore, suggest this species for plantations in the future based on our observation and the local farmers and experts during our research periods. At this age these are likely to be mostly used for fuelwood or pulp [41]. A second harvesting period could occur when the stands are 8–12 years old and a number of species are capable of being large enough for a pole market. Based on species growth rates a third harvesting period would develop around 14–18 years leaving a final harvest of slower growing but higher value trees after 20 years. The selective harvesting could be applied for different species at different times and for different products. Trees of some fast-growing species such as *Leucaena leucocephala, Melia dubia* and *Gmelina arborea* could be early harvested for household consumption or firewood while most of Dipterocarp species could be late harvested for high valued products (e.g. lumber). By this time a number of additional seedlings may have regenerated and begun to growing using light from canopy gaps. These seedlings might even be supplemented by enrichment plantings using species favoured by the market. The plantations could then gradually move to become a selectively managed forest or it could be clear-felled and replanted. Throughout this sequence the timbers produced by the plantations are becoming progressively more valuable. Similarly, the market for these timbers is increasing from a largely local market or on-farm use for fuelwood to a regional or national market for higher value sawlogs.

Finally, these mixtures could also achieve the ecological goal of the Rainforestation Farming project i.e. to achieve a planted forest with a physical structure and species composition and succession similar to the original local rainforest ecosystem after around 14–16 years. A three-storey structural complexity is now beginning to develop these plantings resembling natural rainforests in the area. The changes in density and species compositions in these plantings over time indicated a characteristic often found in uneven-aged forests, old-growth forest or natural forest that is a continuous recruitment and mortality in the forest succession [42]. The ratio of Dipterocarp trees to total trees was approximately 1∶4 across sites, which is similar to the species proportion in the three canopy strata at Mt. Pangasugan in Leyte, the Philippines [29].

## Conclusions

It is clear from our results that the composition and structure of the Rainforestation plantations have changed over time, with the plantations being a highly dynamic system. There has been a decrease in the relative importance of shade-intolerant species, especially exotic species and a corresponding increase in the relative importance of shade-tolerant native species. The design of the species mixtures affected the patterns of species loss in that the very high initial stocking rates resulted in high rates of mortality, mostly among the shade-intolerant species and fruit trees. Other changes in species loss were largely due to ad hoc harvesting decisions by the land owners. We draw on our current results to recommend a modified set of prescriptions for smallholder and community tree plantations. These recommendations include a lower initial stocking rate of 1100 trees per hectare; the proportion of fast growing species should be around 30 to 40 per cent of this initial density; and any fruit trees should only be planted on the plantation margin where more space and light are available for crowns to develop.

## Supporting Information

File S1 **Figure S1. Map of the Rainforestation sites in Leyte province, the Philippines.** The numbers in the map refer to the names of sites. **Figure S2. Size class distributions of species groups in the Rainforestation sites.** Depending on species provenance (i.e. exotic and native) or ecological characteristics of species (i.e. Shade-intolerant, Shade-tolerant and Fruit tree). 1a, 2a & 3a: all size classes of provenance groups; 1b, 2b & 3b: three largest size classes of provenance groups; 4a, 5a & 6a: all size classes of ecological groups; and 4b, 5b & 6b: three largest size classes of ecological groups. **Figure S3. The survival and mortality of the most common species at 80 plots of 18 rainforestation sites in period of 2006–2012.**
(DOCX)

## References

[1] Lamb D, Erskine PD, Parrotta JA (2005) Restoration of degraded tropical forest landscapes. Science 310: 1628. 16339437 10.1126/science.1111773

[2] Debell DS, Cole TG, Whitesell CD (1997) Growth, Development, and Yield in Pure and Mixed Stands of Eucalyptus and Albizia. Forest Science 43: 286–298.

[3] Montagnini F, Gonzalez E, Porras C, Rheingans R (1995) Mixed and pure forest plantations in the humid neotropics: a comparison of early growth, pest management and establishment costs. Commonwealth Forestry Review 74: 306–314.

[4] Piotto D, Víquez E, Montagnini F, Kanninen M (2004) Pure and mixed forest plantations with native species of the dry tropics of Costa Rica: a comparison of growth and productivity. Forest Ecology and Management 190: 359–372.

[5] Kelty MJ (2006) The role of species mixtures in plantation forestry. Forest Ecology and Management 233: 195–204.

[6] Lamb D (2011) Regreening the bare hills: Tropical forest restoration in the Asia-Pacific region; Palo M, editor. Dordrecht: Springer.

[7] Hooper DU, Chapin FS III, Ewel JJ, Hector A, Inchausti P, et al (2005) Effects of biodiversity on ecosystem functioning: A consensus of current knowledge. Ecological Monographs 75: 3–35.

[8] Wormald TJ (1992) Mixed and pure forest plantations in the tropics and subtropics. Rome: FAO. 152 p.

[9] Kelty MJ (1992) Comparative productivity of monocultures and mixedspecies stands. In: Kelty MJ, Larson BC, Oliver CD, editors. The Ecology and Silviculture of Mixed-Species Forests. Dordrecht, Boston: Kluwer Academic Publishers. 125–141.

[10] Haggar JP, Ewel JJ (1997) Primary Productivity and Resource Partitioning in Model Tropical Ecosystems. Ecology 78: 1211–1221.

[11] Bullock BP, Burkhart HE (2005) An evaluation of spatial dependency in juvenile loblolly pine stands using stem diameter. Forest Science 51: 102–108.

[12] Grant JC, Nichols JD, Pelletier M-C, Glencross K, Bell R (2006) Five year results from a mixed-species spacing trial with six subtropical rainforest tree species. Forest Ecology and Management 233: 309–314.

[13] Kooyman RM (1996) Growing rainforest: rainforest restoration and regeneration - recommendations for the humid sub-tropical region of northern New South Wales and south east Queensland. Brisbane: Greening Australia - Queensland. 79 p.

[14] Lamprecht H (1989) Silviculture in the tropics. Tropical forest ecosystems and their tree species - Possibilities and methods for their long-term utilization. Eschborn, Federal Republic of Germany: Technical Co-operation.

[15] Herbohn JL, Vanclay J, Nguyen H, Le HD, Baynes J, et al. (2014) Inventory Procedures for Smallholder and Community Woodlots in the Philippines: Methods, Initial Findings and Insights. Small-Scale Forestry 13: 79–100.

[16] Le HD, Smith C, Herbohn J (2014) What drives the success of reforestation projects in tropical developing countries? The case of the Philippines. Global Environmental Change, 24:334–348.

[17] Le HD, Smith C, Herbohn J, Harrison S (2012) More than just trees: Assessing reforestation success in tropical developing countries. Journal of Rural Studies 28: 5–19.

[18] Nguyen H, Herbohn J, Firn J, Lamb D (2012) Biodiversity–productivity relationships in small-scale mixed-species plantations using native species in Leyte province, Philippines. Forest Ecology and Management 274: 81–90.

[19] Nguyen HTT (2011) Performance of mixed-species plantaitons using the Rainforestation Farming system in Leyte province, the Philippines [PhD thesis]. The University of Queensland.

[20] Milan PP (1997) Appraisal mission of rainforestation farming in Leyte. Baybay, Leyte: Visayas State College of Agriculture (ViSCA). 16 p.

[21] Milan PP (1997) Strategy for community involvement in Rainforestation farming. In: Margraf J, Goltenboth F, Milan PP, editors; 1997 3–6 March; Leyte. Visayas College of Agriculture (ViSCA) - GTZ. 336.

[22] Kolb M (2003) Silvicultural analysis of “Rainforestation Farming” areas on Leyte island, Philippines [PhD thesis]. Deutschland: Universitat Gottingen. 116 p.

[23] Jahn R, Asio VB (2001) Climate, geology, geomorphology and soils of the tropics with special reference to Leyte islands (Philippines). In: Goltenboth F, Asio VB, editors; 2001 9–20 Apr; Baybay, Leyte. Visaya State College of Agroculture. 25–43.

[24] Marohn C (2007) Rainforestation farming on Leyte island, Philippines - aspects of soil fertility and carbon sequestration potential [PhD thesis]. Stuttgart: University of Hohenheim.

[25] Hussain I (2008) Plant Physiology. 1 ed. New Delhi: Oxford Book Co.

[26] Milan PP, Ceniza MJC, Asio VB, Bulayog SB, Napiza MD (2004) Evaluation of silviculltural management, ecological change and market study of products of existing rainforestation demonstration and cooperators’ farms. Baybay: Institute of Tropical Ecology.

[27] Schulte A (1998) Community-based rainforestation options for the Visayas, Philippines: a review. Hoxter, Germany: GTZ.

[28] Schulte A (2002) Rainforestation Farming: Option for rural development and biodiversity conservation in the humid tropics of Southeast Asia. A review of major issues on community-based rehabilitation silviculture and guide to recommended native tree species for the Visayas/Philippines; Goltenboth F, Milan P, editors. Aachen: Shaker. 312 p.

[29] Margraf J, Milan P (1996) Ecology of Dipterocarp forests and its relevance for island rehabilitation in Leyte, Philippines. In: Schulte A, Schone D, editors. Dipterocarp forest ecosystems: Towards sustainable management. Berlin: World Scientific. 124–154.

[30] Barbour MG (1999) Terrestrial plant ecology. Menlo Park, Calif.: Benjamin/Cummings. 1 v. (various pagings).

[31] Barbour MG, Burk JH, Pitts WD (1980) Terrestrial plant ecology. Menlo Park, Calif.: Benjamin/Cummings Pub. Co. xi, 604 p.

[32] Arroyo-Rodríguez V, Mandujano S (2006) The Importance of Tropical Rain Forest Fragments to the Conservation of Plant Species Diversity in Los Tuxtlas, Mexico. Biodiversity & Conservation 15: 4159–4179.

[33] Dangol DR, Shivakoti GP (2001) Species composition and dominance of plant communities in Westren Chitwan, Nepal. Napal Journal of Science and Technology 3: 69–78.

[34] Shrestha R, Karmacharya SB, JHA PK (2000) Vegetation analysis of natural and degraded forests in Chitrepani in Siwalik region of Central Nepal. Tropical Ecology 41: 111–114.

[35] Guariguata M, Chazdon R, Denslow J, Dupuy J, Anderson L (1997) Structure and floristics of secondary and old-growth forest stands in lowland Costa Rica. Plant Ecology 132: 107–120.

[36] Nebel G, Kvist LP, Vanclay JK, Christensen H, Freitas L, et al (2001) Structure and floristic composition of flood plain forests in the Peruvian Amazon: I. Overstorey. Forest Ecology and Management 150: 27–57.

[37] Crane JH (2004) Selected cultural techniques to improve production of some subtropical and tropical fruit crops. In: Albrigo LG, Sauco VG, editors. Acta Horticulturae; 2004 29 Feb 2004; Toronto. 179–187.

[38] Ferguson L, Krueger WH, Reyes H, Metheney P (2002) Effect of mechanical pruning on California black ripe (Olea europea L.) cv. ‘Manzanillo’ table olive yield. In: Vitagliano C, Martelli GP, editors. Proceedings of the Fourth International Symposium on Olive Growing. Leuven. 281–284.

[39] Medina-Urrutia VM, Nunez-Elisea R (1997) Mechanical pruning to control tree size, flowering, and yield of mature “Tommy Atkins” mango trees. In: Lavi U, Degani C, Gazit S, Lahav E, Pesis E et al., editors. 5th International Mango Symposium. Israel. 305–314.

[40] Schupp JR, Baugher TA, Miller SS, Harsh RM, Lesser KM (2008) Mechanical thinning of peach and apple trees reduces labor input and increases fruit size. Horttechnology 18: 660–670.

[41] Tolentino EL (2008) Restoration of Philippine Native Forest by Smallholder Tree Farmers. In: Snelder DJ, Lasco RD, editors. Smallholder Tree Growing for Rural Development and Environmental Services. Dordrecht; London: Springer. 319–346.

[42] Oliver CD, Larson BC (1996) Forest stand dynamics. Updated edition. Wiley, New York.

