# Correction: Designing Mixed Species Tree Plantations for the Tropics: Balancing Ecological Attributes of Species with Landholder Preferences in the Philippines

**DOI:** 10.1371/journal.pone.0098600

**Published:** 2014-05-20

**Authors:** The PLOS ONE Staff

The species names for *Casuarina equisetifolia* and *Acacia* sp. are misspelled in the Discussion section.

The last row of Table 6 is incorrect. Please see the corrected table below.

**Table 6:** Recommended design for smallholder tree plantations in Leyte, Philippines based on performance of Rainforestation Farming plantations

Product	Time of thinning (yrs)	Number of species	Density (trees/ha)	Typical species
Firewood	6-10	3-5	450	Gmelina (*Gmelina arborea*)
				Bagalunga (*Melia dubia*)
				Ipil-Ipil (*Leucaena laucocephala*)
				Raintree (*Samanea saman*)
				Mt agoho (*Gymnostoma rumphianum*)
				Agoho (*Casuarina equisetifolia*)
				Thailand acacia (*Senna siamea*)
Pole	8-12	2-3	200	Gmelina (*Gmelina arborea*)
				Mahogany (*Swietenia macrophylla*)
				Kalumpit (*Terminalia macrocarpa*)
				Dao (*Dracontamelon dao*)
				Narra (*Pterocarpusindicus*)
				Mt agoho (*Gymnostoma rumphianum*)
Fast-growing timber	14-18	3-5	250	Gmelina (*Gmelina arborea*)
				Mahogany (*Swietenia macrophylla*)
				Teak (*Tectona grandis*)
				Mayapis (*Shorea palosapis*)
				White lauan (*Shorea contorta*)
				Almaciga (*Agathis philippinensis*)
				Tangeli (*Shorea polysperma*)
				Bagtikan (*Parashorea plicata*)
Slower-growing timber	> 20	3-10	200	Yakal saplungan (*Hopea plagata*)
				Yakal kaliot (*Hopea malibato*)
				Malakawayan (*Podocarpus rumphii*)
				Molave (*Vitex parviflora*)
				Narra (*Pterocarpus indicus*)
				Malapanau (*Dipterocarpus kerrii*)
				Tangeli (*Shorea polysperma*)
				Apitong (*Dipterocarp grandiflorus*)
				Apitong hagakhak (*Dipterocarpus kunstleri*)
				Dalingdingan (*Hopea dalingdingan*)
				Red lauan (*Shorea negrosensis*)
				Bitanghol (*Calophyllum blancoi*)
				Bitanghol sibat (*Calophyllum lancifolium*)
Total		11-23	1100	
Fruit trees that could be planted on plantation margin				Durian (*Durio zibethinus*), Mango (*Mangifera indica*), Rambutan (*Nephelium lappaceum*), Nangka (*Artocarpus heterophyllus*)
