# Supplementary figures and images for: Designing Mixed Species Tree Plantations for the Tropics: Balancing Ecological Attributes of Species with Landholder Preferences in the Philippines
Source: PLoS One. 2014 Apr 21;9(4):e95267. doi: 10.1371/journal.pone.0095267 (PMC3994060; doi:10.1371/journal.pone.0095267)

**Supporting information**


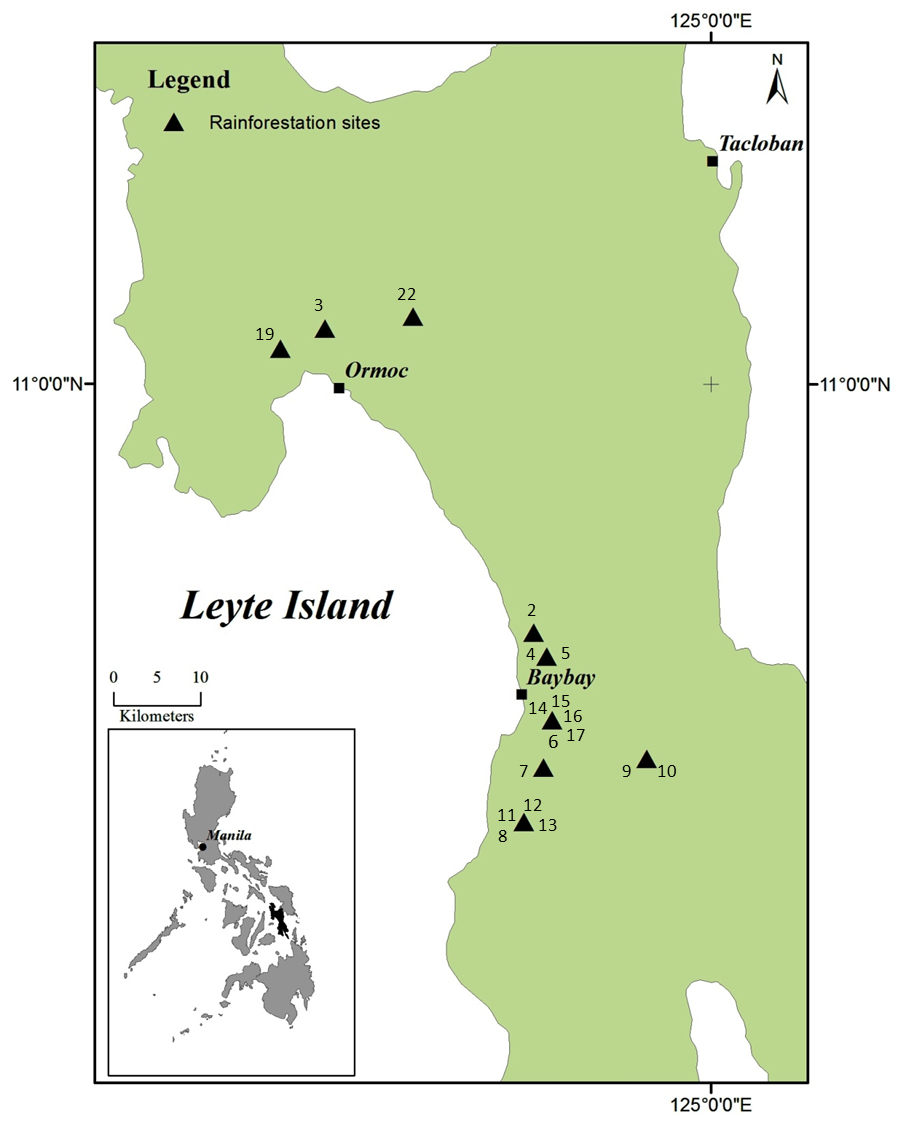


**Figure S1.**

**Figure S2.**


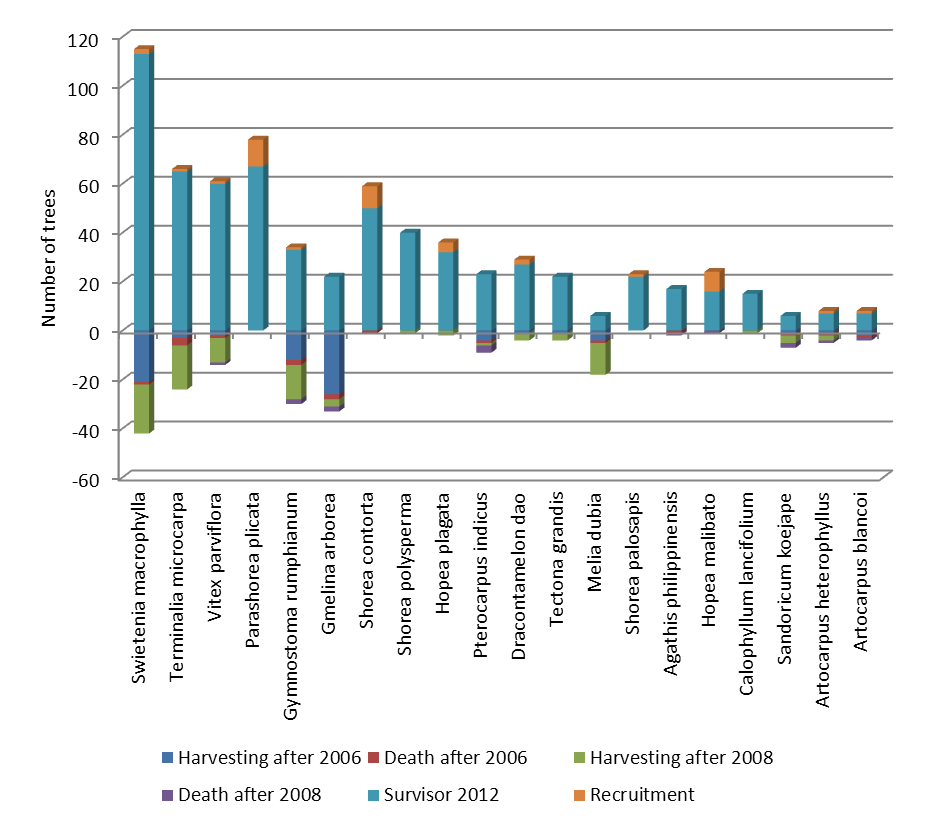


**Figure S3.**

Supplement: File S1 — Figure S1. Map of the Rainforestation sites in Leyte province, the Philippines. The numbers in the map refer to the names of sites. Figure S2. Size class distributions of species groups in the Rainforestation sites. Depending on species provenance (i.e. exotic and native) or ecological characteristics of species (i.e. Shade-intolerant, Shade-tolerant and Fruit tree). 1a, 2a & 3a: all size classes of provenance groups; 1b, 2b & 3b: three largest size classes of provenance groups; 4a, 5a & 6a: all size classes of ecological groups; and 4b, 5b & 6b: three largest size classes of ecological groups. Figure S3. The survival and mortality of the most common species at 80 plots of 18 rainforestation sites in period of 2006–2012. (DOCX) [file pone.0095267.s001.docx]
